# Aminopropyl-Silica Hybrid Particles as Supports for Humic Acids Immobilization

**DOI:** 10.3390/ma9010034

**Published:** 2016-01-08

**Authors:** Mónika Sándor, Cristina Lavinia Nistor, Gábor Szalontai, Rusandica Stoica, Cristian Andi Nicolae, Elvira Alexandrescu, József Fazakas, Florin Oancea, Dan Donescu

**Affiliations:** 1 Faculty of Chemistry and Chemical Engineering, Babes-Bolyai University, Arany János Street, No.11, Cluj-Napoca 400028, Romania; sandormonika16@gmail.com (M.S.); fazakaseniko7@gmail.com (J.F.); 2 National Research & Development Institute for Chemistry and Petrochemistry—ICECHIM, Splaiul Independentei No.202, 6th district, PO Box 35-174, Bucharest 060021, Romania; rusandica.stoica@gmail.com (R.S.); cristian.nicolae@icechim-pd.ro (C.A.N.); elvira.alexandrescu@icechim-pd.ro (E.A.); florino@ping.ro (F.O.); ddonescu@chimfiz.icf.ro (D.D.); 3 NMR Laboratory, Institute of Materials Engineering, University of Pannonia, Egyetem Street, No. 10, Veszprém 8200, Hungary; szalontai.gabor@solidnmr.hu

**Keywords:** aminosilane supports, humic acids immobilization, silica hybrid particles, sol-gel process

## Abstract

A series of aminopropyl-functionalized silica nanoparticles were prepared through a basic two step sol-gel process in water. Prior to being aminopropyl-functionalized, silica particles with an average diameter of 549 nm were prepared from tetraethyl orthosilicate (TEOS), using a Stöber method. In a second step, aminopropyl-silica particles were prepared by silanization with 3-aminopropyltriethoxysilane (APTES), added drop by drop to the sol-gel mixture. The synthesized amino-functionalized silica particles are intended to be used as supports for immobilization of humic acids (HA), through electrostatic bonds. Furthermore, by inserting beside APTES, unhydrolysable mono-, di- or trifunctional alkylsilanes (methyltriethoxy silane (MeTES), trimethylethoxysilane (Me_3_ES), diethoxydimethylsilane (Me_2_DES) and 1,2-bis(triethoxysilyl)ethane (BETES)) onto silica particles surface, the spacing of the free amino groups was intended in order to facilitate their interaction with HA large molecules. Two sorts of HA were used for evaluating the immobilization capacity of the novel aminosilane supports. The results proved the efficient functionalization of silica nanoparticles with amino groups and showed that the immobilization of the two tested types of humic acid substances was well achieved for all the TEOS/APTES = 20/1 (molar ratio) silica hybrids having or not having the amino functions spaced by alkyl groups. It was shown that the density of aminopropyl functions is low enough at this low APTES fraction and do not require a further spacing by alkyl groups. Moreover, all the hybrids having negative zeta potential values exhibited low interaction with HA molecules.

## 1. Introduction

Hybrid organic/inorganic materials derived through sol-gel processing have been extensively studied and reviewed [1,2]. These hybrid materials combine the properties of inorganic (e.g., heat resistance, retention of mechanical properties at high temperature, and low thermal expansion) and of organic (e.g., flexibility, low dielectric constant and ductility) compounds in one material [2]. The reactive organosilane compounds have been used for modifying and tailoring the surface properties of inorganic materials, thereby the material will contain the desired functional groups on its surface.

Mono-, di- and trifunctional organosilanes were used as reactive compounds for functionalization, having one, two or three hydrolysable groups [3]. Depending on the reaction conditions, chemistry of the organosilane, and surface history, a number of different structures can be produced on the surface [3,4,5].

The attachment of humic acids (HA) to the silica hybrid particles can lead to the formation of materials with very interesting properties. It is well known that, in soil, humic acids are important chelators, combining minerals into organic compounds that are more available to plants. They also tie up toxins, making them less available to plants. Thus, remediation capacity of HA immobilized on silica surfaces was already investigated in previous studies [5,6,7,8,9,10,11,12,13,14]. It was shown that these HA-SiO_2_ hybrids are effective for remediation of water contaminated with aromatic hydrocarbons [7,11,14], metal ions [6,8,12,13] or radioactive iodine [6,8]. These can also be used to reduce the amount of chemical oxygen demand in wastewater [14]. The aminopropyl functionalized and humic substances activated silica particles have also been used for preparation of supports to immobilize enzymes, which can be widely used in analytical chemistry, and the food, pharmaceutical and chemical industries [15].

Despite the wide variety of silane derivatives, available for surface modification, the majority of studies concerning immobilization of HA on silica supports have employed aminosilanes, in particular 3-aminopropyltriethoxysilane (APTES), because of its intrinsic chemical properties [9,16]. After grafting the amino functional groups in organic solvents, it was found that not all Si-OH groups were derivatized with amino-alkoxysilanes [6,8]. For this reason, there have been attempts to block the -OH residual groups with reactive compounds having simple substituents such as trimethylchlorosilane [6,8].

A similar phenomenon was observed in the case of the immobilization of humic acid on aminopropyl-silica, where only a fraction of the amino groups reacted with the humic acid molecules, due to the steric hindrance [9,10]. The remaining amino groups were blocked with small substituents such as acetic anhydride [9] and sodium acetate [10].

Recent studies related to increasing of interaction efficiency to organically derivatized silica, have shown that this can be achieved by incorporating non-reactive silanes [17]. The capacity of functionalization with trialkoxysilane [5,6,7,8,9,10] or monoalkoxysilane derivatives was previously studied [10]. But, in all the reported situations, derivatization process was performed in organic solvents after a careful dehydration.

The present work is focused on preparation and characterization of a series of aminosilica hybrid particles, prepared by sol-gel technique in aqueous medium, which can be used as supports for humic acids (HA) immobilization. Furthermore, we investigated the effect of grafting, beside 3-aminopropyltrimethoxysilane (APTES), of mono-, di- and trifunctional alkylsilanes, in order to ensure a better access of the large HA molecules to the free amino groups. Thus, the resulted aminosilane supports contain, on one hand, aminopropyl chains with free NH_2_ groups, acting like coupling agents for the humic acids molecules, and, on the other hand, alkylsilane derivatives, as spacers for the -NH_2_ groups. Two sorts of humic acids (a commercial product from Aldrich (St. Louis, MO, USA) (noted here AHA) and a lab-made humic acid, extracted from peat (noted here PHA)) were used for evaluation of the efficiency of coupling on the newly synthesized aminosilane supports.

## 2. Results and Discussions

### 2.1. CHN Elemental Analysis (Determination of the Mass Fraction of Carbon, Hydrogen and Nitrogen)

The carbon percentage contents are listed in Table 1 and vary according to the precursor’s molar ratio used in the sol-gel process. Obviously, the highest carbon content was measured for silica particles synthesized only from APTES (sample 1), while the lowest, for the particles containing only TEOS in the sol-gel mixture (sample 2), Grafting of APTES on the preformed SiO_2_ particles (sample 3) increases the carbon weight share in the final hybrid.

For samples in which, besides TEOS and APTES, alkyl-derivatives were also added to the sol-gel reaction (samples 4–8), the carbon content is slightly lower. Between them, the highest carbon content was found in the Me_2_-DES modified sample5. Grafting of the different alkyl derivatives (methyl, dimethyl, trimethyl) on the surface of silica particles lowers the weight share of aminopropyl groups in the whole reaction system, leading to lower carbon content. These results were also further confirmed by the thermal analyses, the weight loss decreasing for the TEOS/APTES/alkyl silane samples.

**Table 1:** Composition of the different silica systems and the corresponding carbon content and weight loss.

Samples	Silica Systems	Molar Ratio	Carbon (%)	Weight Loss 25–250 °C (%)	Weight Loss 250–450 °C (%)	Weight Loss 450–700 °C (%)	Inorganic Residue at 700 °C (%)
**1**	APTES	-	27.63	23.1	7.5	22.1	47.3
**2**	TEOS	-	0.94	9.6	2.3	2.3	85.8
**3**	TEOS/APTES	20/1	5.98	11.0	4.4	4.1	80.5
**4**	TEOS/APTES/MeTES	20/1/1	3.27	9.6	3.7	2.9	83.8
**5**	TEOS/APTES/Me_2_DES	20/1/1	4.74	9.4	3.5	3.0	84.1
**6**	TEOS/APTES/Me_3_ES	20/1/1	3.83	11.0	3.6	3.2	82.2
**7**	TEOS/APTES/BETES	20/1/0.5	4.42	8.5	3.5	2.7	85.3
**8**	TEOS/APTES/Me_3_ES	10/1/1	5.01	13.6	4.6	5.1	76.7
**9**	TEOS/BETES	10/0.5	2.96	10.6	4.1	2.2	83.1

### 2.2. Thermogravimetric Analysis (TGA)

The TGA results were consistent with previously presented data; the weight loss being in general proportional to the carbon content, the observed small deviations being caused by the removal of the adsorbed water in the first temperature range. Sample 1 (only APTES particles), with the highest carbon content, showed the highest value of weight loss, while for sample 2 (only TEOS particles), having the lowest carbon content, was showed the lowest value of the weight loss.

**Figure 1:**
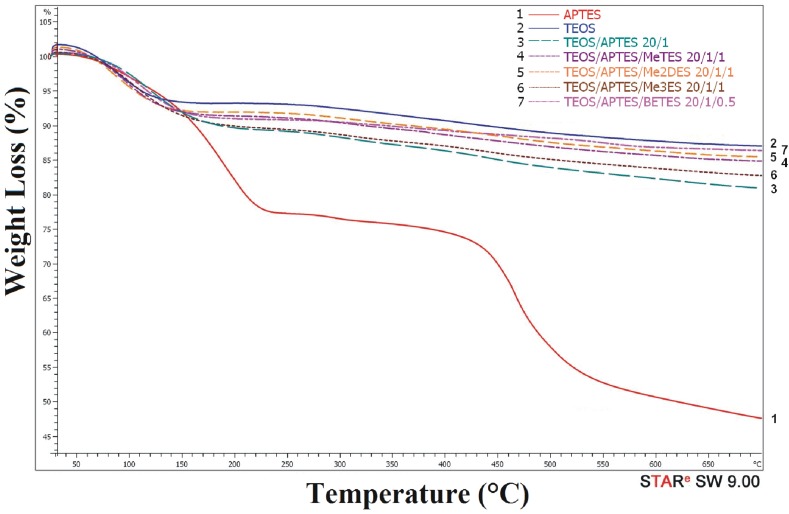
Thermogravimetric (TG) curves of aminopropyl-silica particles.

Figure 1 shows the TG profiles of different aminopropyl functionalized silica systems. The most important differences were observed in the first temperature range (25–250 °C), which corresponds to the release of water and other solvents. Having a hydrophilic character and a less cross-linked network, sample 1 shows the highest weight loss due to water removal.

The second temperature range (250–450 °C) was due to the degradation of aminopropyl and of alkyl chains (when alkyl silanes were added to the sol-gel system). The third temperature range (450–700 °C) corresponds to dehydroxylation of Si-OH residual functions, which led to the formation of Si-O-Si groups. Sample 1 (only APTES), which has a bulky aspect after drying, exhibits a larger weight loss due to the higher content of uncondensed Si-OH functions.

From thermogravimetric analysis it was shown that the thermal behavior of silica hybrids was affected by the nature and concentration of the used organosilanes. Thus, the value of the inorganic residue measured at 700 °C decreased accordingly to the increase in the content of organic compounds (Table 1).

### 2.3. Particle Size Measurements (Dynamic Light Scattering (DLS) Technique)

In Table 2 are presented the values of the average hydrodynamic diameters recorded for samples diluted in distilled water. For sample 1, the measured average particle diameter is 580 nm. The measurement precision for this sample was lower because the sol dispersion was very complex, containing aggregates of various sizes. After the drying of this sol-gel system a bulky material was obtained (see later the Environmental Scanning Electronic Microscopy (ESEM) results). Sample 2 has an average diameter of 549 nm and a narrow size distribution, indicating the successful synthesis of uniform Stöber silica particles (see later the ESEM images).

**Table 2:** Particle size and zeta potential values for the studied supports.

Samples	Silica Systems	Molar Ratio	Average Diameter (nm)	Zeta Potential (mV)
**1**	APTES	-	580	−19
**2**	TEOS	-	549	−45
**3**	TEOS/APTES	20/1	691	15
**4**	TEOS/APTES/MeTES	20/1/1	1471	52
**5**	TEOS/APTES/Me_2_DES	20/1/1	858	27
**6**	TEOS/APTES/Me_3_ES	20/1/1	653	13
**7**	TEOS/APTES/BETES	20/1/0.5	716	53
**8**	TEOS/APTES/Me_3_ES	10/1/1	558	−5
**9**	TEOS/BETES	10/0.5	590	−48

According to the DLS measurements, by grafting the aminopropyl functions on the surface of preformed silica particles, the hydrodynamic diameter of the resulted particles increases from 549 (sample 2) to 691 nm (sample 3). When alkyl derivatives have been used, the particles size depends on the addition of alkyl-silanes beside APTES in the reaction system.

**Figure 2:**
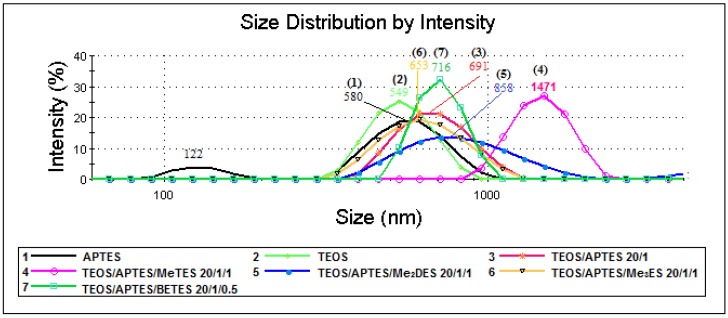
Size distribution by intensity of aminopropyl silica particles.

When BETES is used as spacer (sample 7), the recorded average diameter (716 nm) is just slightly higher than the one recorded for sample 3 (691 nm). Though, a narrower size distribution is shown for sample 7 when compared with sample 3, being already well known that BETES acts as a cross-linking agent in the sol-gel reaction, leading to a more compact silica network (Figure 2). It also should be noted that, by introduction of the methyl or dimethyl spacers, the size increases significantly and the distribution becomes much broader. This is probably due to the increased ability of particles to form aggregates due to the hydrophobic interaction induced by the methyl groups (Figure 2). Therefore, the highest values of the average hydrodynamic diameter (Table 2) were registered for samples 4 (1471 nm) and 5 (858 nm), respectively. These two samples showed a very hydrophobic behavior, floating on the top of the aqueous dispersion and requiring an increased effort to be homogenized for the DLS analyze.

A surprising result was obtained for sample 6 (where Me_3_ES has been used as spacer) for which both the average diameter (653 nm) and size distribution are almost similar to that obtained for sample 3 (691 nm), indicating, in agreement with the C-13 ssNMR (solid state Nuclear Magnetic Resonance) data (see later), the absence of integration of thetrimethyl derivative in the formed silica network.

### 2.4. Zeta Potential Measurements (Laser Doppler Velocimetry (LDV) Technique)

**Figure 3:**
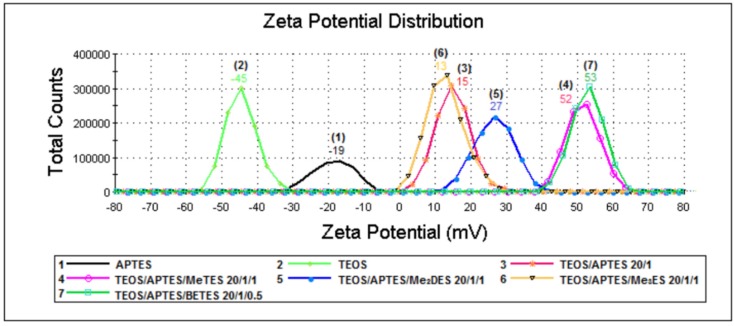
Zeta potential distribution of aminopropyl silica particles.

In Figure 3, zeta potential values (the potential that exists at the particle’s hydrodynamic share or slipping plane boundary) are illustrated for samples dispersed in distilled water. As we expected, for samples 2 and 9, the value of zeta potential is highly negative (−45 mV and −48 mV, respectively), due to the high density of residual Si-OH present on the surface of silica particles.

By grafting the aminopropyl functions onto the surface of the preformed TEOS silica particle, the surface charge of particle was modified. As a result, the value of zeta potential shifts towards positive values (samples 3–7, Table 2). In addition, the alkyl-aminopropyl hybrids (samples 4–7) show an increase of zeta potential values, with the increase of the probability of interaction of the Si-OH from the particles surface with the available reactive ethoxy groups from the alkyl-silanes. In these conditions, in distilled water, the -NH_2_ functions from APTES can be protonated in an increasingly higher extent. Thus, co-grafting (beside APTES) of di- or tri-functional alkyl-silanes (MeTES—sample 4 and BETES—sample 7) led to the highest values of zeta potential (+52 and +53 mV, respectively). The hybrid with the bi-functional co-precursor (Me_2_DES—sample 5) exhibited a lower zeta potential (+27 mV), while the use of the mono-functional co-precursor (Me_3_ES—sample 6) led to a value of zeta potential (+13 mV) very similar with the one recorded for the hybrid prepared with APTES and TEOS only (sample 3, +15 mV).

As mentioned above (Section 2.3), for samples 4 and 5 a hydrophobic behavior was noticed (the silica powder was floating). This change of the hybrid properties proves the structure modification of the surface of the reference APTES/TEOS hybrid (sample 3). When Me_3_ES was used as co-precursor beside TEOS and APTES (sample 6), the expected hydrophobic behavior, thought to be given by the insertion of the trimethyl functions in the final hybrid network, was not noticed anymore. All these observations come to strengthen the idea that Me_3_ES does not efficiently connect to the silica network.

For samples 1 and 8, by significantly increasing the aminopropyl density, this hydrophobic chains tend to self-associate and to orientate towards the interior of silica particles. A phase-inversion mechanism occurs, the -NH_2_ functions being mostly hidden inside the silica hybrid frame, while towards water remain predominantly exposed Si-O charges. Thus, for the both samples, the value of zeta potential remains negative (−19 mV and −5 mV, respectively), but less negative than the one recorded for samples without APTES, since the amino functions are still partially able to interact with the surrounding medium.

### 2.5. Environmental Scanning Electronic Microscopy (ESEM)

**Figure 4:**
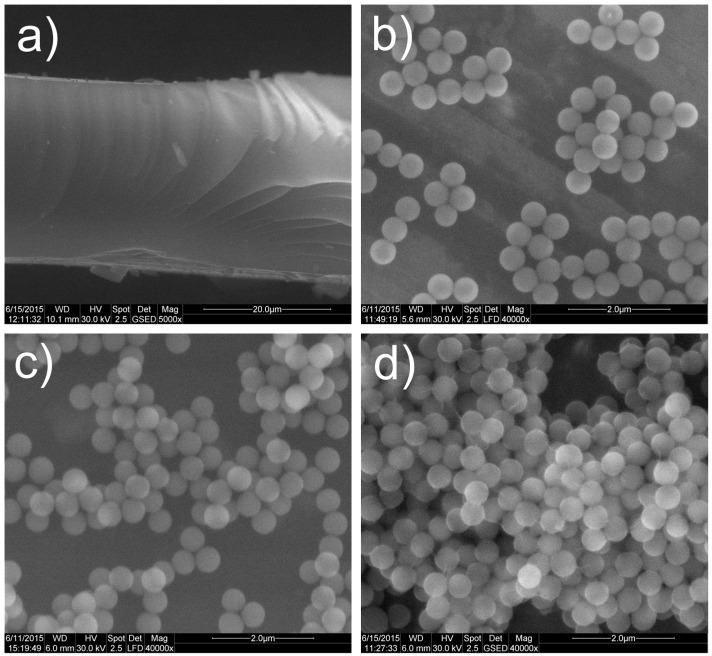
ESEM images of: (**a**) APTES film (Sample 1); (**b**) pristine TEOS silica particles (Sample 2); (**c**) APTES-grafted silica particles (Sample 3); and (**d**) MeTES and APTES—grafted silica particles (Sample 4).

ESEM images of some of the studied aminosilica hybrids are shown in Figure 4. Sample 1, obtained from the sol-gel reaction of APTES only, is a film forming hybrid, of which cross-section can be seen in Figure 4a. In Figure 4b the uniform and spherically shaped SiO_2_ particles resulted from the TEOS reaction (sample 2) are clearly shown. By grafting a small amount of APTES on these preformed SiO_2_ particles (1 mole of APTES at 20 moles of TEOS), no significant change in the particles shape or size could be seen (Figure 4c).

Unlike the hydrodynamic diameter (measured by DLS technique—see Section 3.3), which was measured in an aqueous dispersion, where the organic chains from the particle’s surface were found in an extended state and where aggregates were allowed to form and to affect the measurement’s result, the particles size estimated by ESEM analyze seems to not change after grafting the aminopropyl or alkyl chains onto particle’s surface. Thus, for sample 4 (Figure 4d), a closer packing of the methyl- and aminopropyl-functionalized particles is noticed due to the hydrophobic interactions. However, the size of the individual particles seems unchanged after drying.

### 2.6. Fourier Transformed Infrared Spectroscopy (FTIR) Spectra

**Figure 5:**
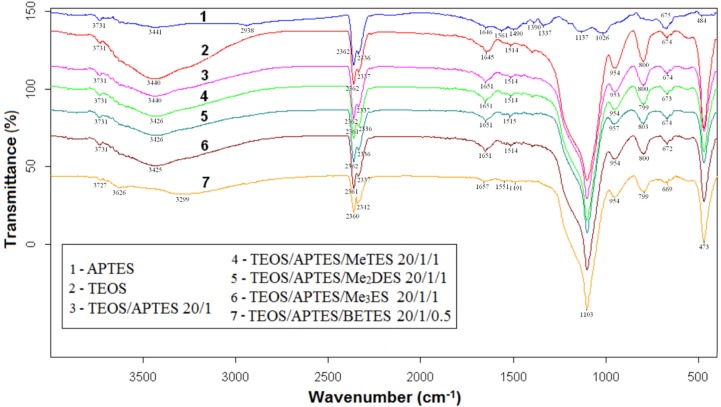
FTIR spectra of aminopropyl functionalized silica particles.

Figure 5 shows the FTIR spectra in the region 4000–400 cm^−1^ of the pristine TEOS silica and of aminopropyl-grafted silica hybrids. Because APTES used for functionalization of the silica particles have a very small weight share compared with TEOS used to obtain the initial particles (TEOS/APTES = 20/1 molar ratio), its specific vibrations are overlapped by the stronger vibrations of the inorganic silica network.

Thus, the broad band covering the range from 3000 to 3800 cm^−1^ can be assigned to hydrogen bonded and partially hydrated silanols (Si-OH). This band was caused by overtones or combinations of vibrations of Si-OH or H_2_O and is generally composed of stretching modes.

For samples grafted with aminopropyl, the silanol groups that are shown in the spectrum of sample 2 (the broad band at ~3440 cm^−1^) were partially consumed by reaction with APTES.

The region of 3000–2800 cm^−1^ corresponds to symmetrical and asymmetrical stretching vibrations of CH_2_ from aminopropyl segment. In 1680–1620 cm^−1^ region, the band at ~1630 cm^−1^ characteristic to NH_2_ deformation in primary amines (due to the formation of NH_3_^+^ as the carbon dioxide is adsorbed onto the silica hybrid), is overlapped by the band at ~1650 cm^−1^, assigned to C=O stretching in carbonyl compounds. Peak 1491 cm^−1^ was attributed to N-H vibration (bending mode) in the primary amine group (R-NH_2_). The band at 1390 cm^−1^ have been assigned to the bicarbonate species (C=O bending). In the presence of water, one mole of amine can chemisorb one mole of CO_2_, leading in the end to the formation of a bicarbonate group [18,19,20].

The main bands in the region 1300–400 cm^−1^ are associated with the combination of vibrations of silica network: the strong band that emerged at ~1103 cm^−1^ (SiO_4_ ring asymmetric stretching), ~954 cm^−1^ (Si-O-Si symmetric stretching within the SiO_4_ tetrahedron), ~800 cm^−1^ (O-Si-O symmetrical stretching), and ~473 cm^−1^ (Si-O-Si bending in SiO_4_ tetrahedron). As it was expected, the intensities of these absorption bands decrease with reducing the weight percentage of TEOS in the final hybrid.

### 2.7. Solid State Nuclear Magnetic Resonance (ssNMR)

Valuable structural information about the obtained amino-silica supports can be obtained by means of ^29^Si [21] and ^13^C [22] ssNMR. It also proved extremely useful in characterization of sol-gel-derived materials [23]. This technique has been used under the condition of cross polarization (CP) and magic angle spinning (MAS) [24] for structural characterization of silica particles used. In general, in the CP/MAS spectra, the Q^4^groups are heavily underrepresented [4].

**Figure 6:**
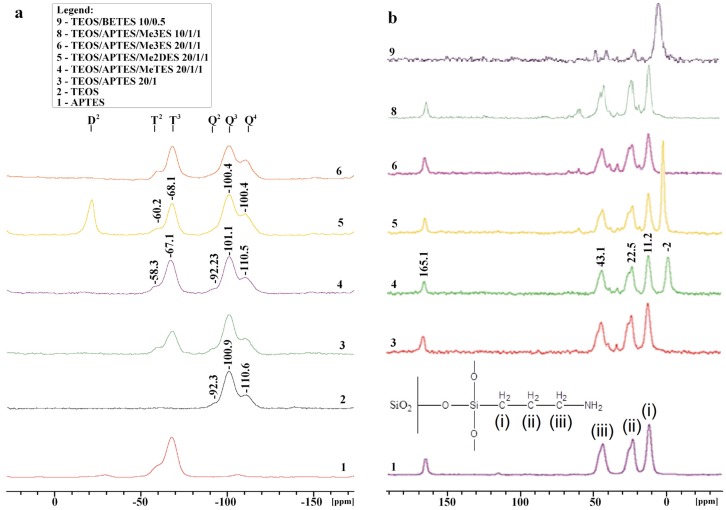
(**a**) ^29^Si CP/MAS–NMR; and (**b**) ^13^C CP/MAS–NMR solid state spectra of pristine and modified silica particles.

The ^29^Si NMR spectra of un-grafted (pristine) SiO_2_ particles (sample 2) and hybrid silica particles (samples 3–6) are shown in Figure 6a. The assignments of the signals to the D, T and Q sites [21] are also shown. The spectrum of sample 2 contains three signals at chemical shifts of −92.3, −100.9, and −110.6 ppm, which have been, respectively, assigned to (i) silicon atoms with two attached hydroxyl groups, designated as Q^2^; (ii) silicon atoms with one attached hydroxyl groups, designated as Q^3^; and (iii) silicon atoms with no hydroxyls, designated as Q^4^.

The attachment of di- and trifunctional co-precursors leads to the appearance of extra signals. Thus, signal at −20 ppm is assigned to -O_2_SiR_2_ structures, designated as D^2^, the signal around −60 ppm is assigned to -O_2_Si(OH)R structures, designated as T^2^, and signal around −68 ppm is assigned to -O_3_SiR structures, designated as T^3^. The signal at about −20 ppm, shown in the sample 5 spectrum, is due to the covalently attached Me_2_DES molecules (a D^2^ group), while the T^2^ and T^3^ signals indicate the attachment of the hydrolyzed APTES (and MeTES for sample 4) to the preformed TEOS-based silica network. Addition of monofunctional ligands (e.g., Me_3_ES) could not be confirmed (see the spectrum of sample 6, which is identical to that of sample 3).

The spectrum of sample 1 (only APTES-based homogenous bulky material), apart from the spinning side bands, contains the same T signals as the APTES-grafted SiO_2_ particles (samples 3–6). As expected, the spectrum exhibits only trifunctional Si atoms, thus here we are facing with an irregular (random) or one of the possible cage structures formed in a self-condensation process.

Figure 6b shows the ^13^C CP/MAS spectra obtained for APTES or TEOS/APTES hybrids, (spectra 1 and 3–8). In the ^13^C NMR spectrum of sample 1 three signals are seen at 43 ppm (shoulder at 45.6), 22.5 ppm (shoulder at 25.2) and 11 ppm in accordance with the expectations. Note, however, that the two high-frequency signals consist of two overlapping peaks. This suggests that carbons (ii) and (iii) (see Figure 6b) exist in two different chemical environments. In this respect, the presence of the 165.1 ppm signal in spectrum of all APTES derivatives gives some clue. Leal *et al.* [18] showed that the adsorption of CO_2_ on the aminopropyl-modified silica material is due to the formation of carbamate species, where two amino groups from the silica surface are used up per carbon dioxide adsorbed molecule. If so, the 165.1 ppm signal can be assigned to a carbamate carbon atom [25].

For samples 4 (TEOS/APTES/MeTES) and 5 (TEOS/APTES/Me_2_DES), the appearance of a new signal at −2.3 and 1.7 ppm, assigned to the methyl groups, indicates the integration of the hydrolyzed mono- and di-alkyl silica derivatives in the TEOS-based silica network, respectively. However, regardless of the ratio used, the more bulky monofunctional silanes (Me_3_ES, samples 6 and 8) did not bind to the silica surface (Figure 6b). In a control sample, prepared only from TEOS and BETES (sample 9), the presence of Si-CH_2_- groups was detected (see the strong signal at 1.7 ppm), while the 165.1 ppm carbonyl signal was no longer observed. This can be taken as further confirmation that only the amino-functionalized hybrids (all APTES containing samples) have the ability to capture the atmospheric CO_2_, unlike the ligands without the terminal amine group, such as the TEOS/BETES hybrid.

### 2.8. Thermogravimetric Analysis Coupled with Mass Spectroscopy (TG-MS)

**Figure 7:**
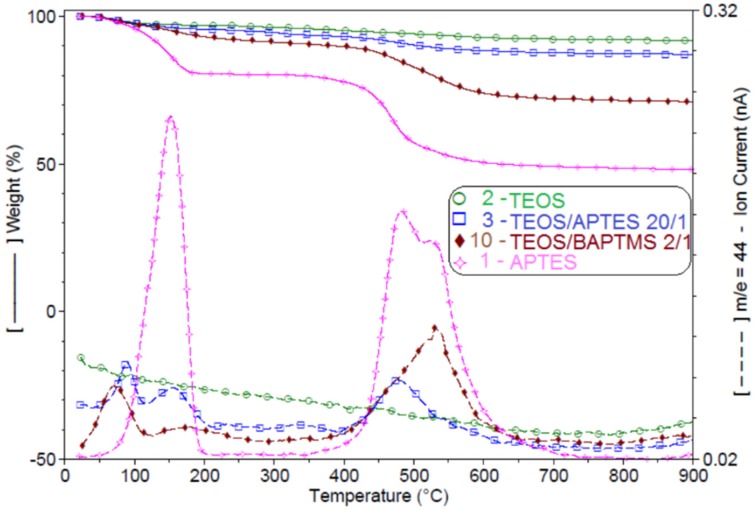
CO_2_ weight loss (TG-MS diagrams) recorded for representative aminosilica supports.

The contamination/reaction with atmospheric CO_2_ of amino-functionalized hybrids was evaluated by TG-MS analysis. The weight loss for the examined samples during controlled heating (following a specific heating profile) is represented in Figure 7. The largest weight loss in the interval 0–200 °C (Figure 1) was observed for sample 1, obtained through sol-gel reactions of APTES only and which contains about twenty times more NH_2_ groups than the other samples. In the same temperature interval, CO_2_ is eliminated from all samples except sample 2.

When compared to sample 1, sample 3 shows a lower CO_2_ weight loss as it has a much lower density of amino groups. A lower CO_2_ weight loss was also measured for sample 10 (a control sample synthesized from TEOS/bis(aminopropyl)trimethoxysilane (BAPTMS) 2/1).

Containing secondary amines the BATMS/TEOS hybrid exhibits a lower reactivity towards CO_2_. In 0–200 °C interval, the weight loss recorded for samples 3 and 10 takes place in two steps, at temperatures below and above 100 °C. One possible explanation is that a part of CO_2_ is physically adsorbed on the surface (is removed in the first step at about 85 °C) and another part is chemically bounded (is lost in the second step at about 165 °C). The CO_2_ weight loss between 400 and 600 °C can be due to the thermo-oxidative decomposition of the organic groups found in the final hybrids.

### 2.9. Porosimetry

**Figure 8:**
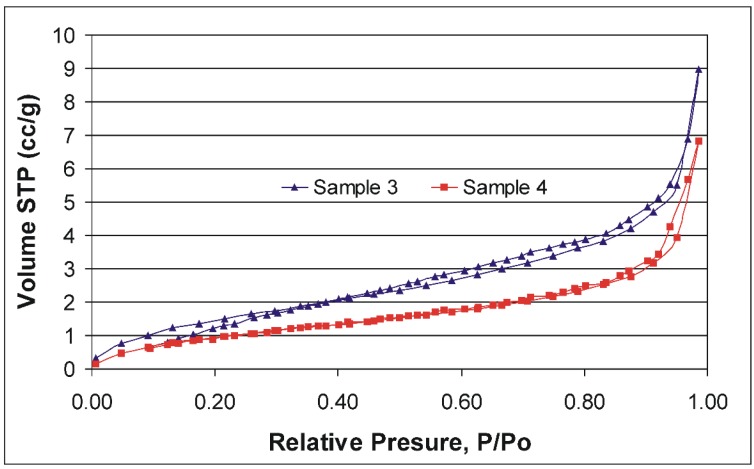
Nitrogen adsorption-desorption isotherms for two selectedaminopropyl supports.

Figure 8 compares the nitrogen adsorption-desorption isotherms of two selected aminosilica supports (samples 3 and 4). The resulted isotherms can be assigned as type II, according to the IUPAC classification. The observed hystereses are due to the presence of textural pores (inter-particles pores). As expected, the BET (Brunauer–Emmett–Teller) specific surface areas recorded for the two samples are very small (6 m^2^/g for the both studied materials) and so is the total pore volume (0.01642 cm^3^/g for sample 3 and 0.01389 cm^3^/g for sample 4). Thus, our aminopropyl-grafted SiO_2_ particles are not mesoporous and it is unlikely that the large and complex HA molecules can be adsorbed in the particle’s cavities.

### 2.10. The Humic Acid (HA) Immobilization Tests of the Novel Aminosilica Supports

**Figure 9:**
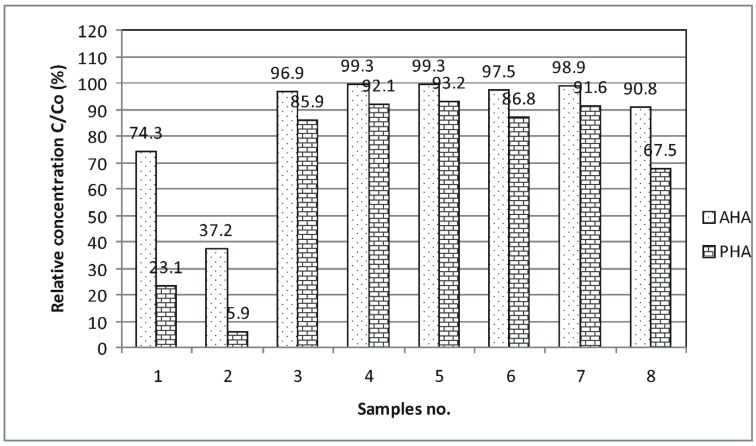
Relative Concentration of immobilized humic acids (AHA and PHA, respectively) as a function of silica support type.

Two sorts of humic acids—AHA and PHA—were selected for testing the functionalization efficiency of the synthesized aminosilica hybrids. The relative concentrations of HA immobilized on various silica supports are represented in Figure 9. Obviously, the lowest concentration of up-taken HA was determined for sample 2, having only Si-OH groups on the surface. Sample 1, synthesized only from APTES, shows an almost twice higher concentration of chemically immobilized HA if compared with sample 2, due to its amino functions. In previous studies [9,10] it was shown that the large molecules of humic acids are sterically hindered to attach to the amino functions of the aminosilica supports. More than that, through zeta potential measurements, we showed above (see Section 2.4.) that a significant amount of the amino functions are hidden inside the silica frame resulted from self-condensation of APTES, while towards the exterior remains mostly Si-O ions, less reactive with HA molecules. Therefore, it is not surprising that for the hybrid (sample 3) (TEOS/APTES 20/1), which has a much lower density of the aminopropyl groups on the surface than sample1 and has a positive value of zeta potential (most of the amino functions are located toward the exterior of the silica particles), the HA-binding ability is greatly improved.

**Figure 10:**
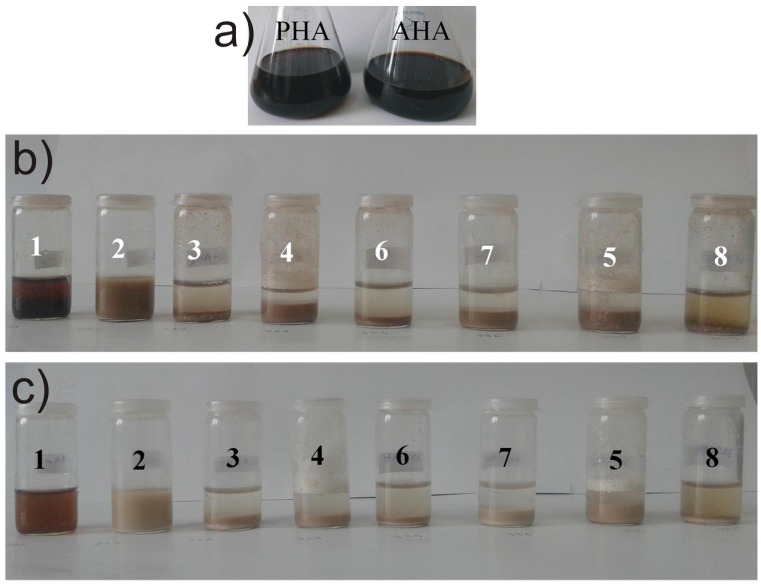
(**a**) Pictures of the two humic acid stock solutions and immobilization tests of (**b**) AHA; and (**c**) PHA, respectively, on various silica supports.

Despite our expectations, for samples where methyl-, dimethyl- or ethyl-groups were used as spacers for the amino-functions (samples 4, 5 and 7), the improvements of the immobilization capacity are not impressive enough (~2% for AHA and ~6% for PHA immobilization) to justify the usefulness of aminopropyl spacing by alkyl groups. So, the density of aminopropyl functions seem to be small enough at this low APTES molar ratio (TEOS/APTES = 20/1) not to sterically hinder the interaction with the HA molecules. Further spacing by alkyl groups is not therefore an absolute need (see also Figure 10). In addition, the inefficient grafting of Me_3_ES co-precursor on the silica hybrid network is also confirmed by the coupling tests, in which similar (sample 6) or lower concentration (sample 8) of immobilized HA was determined compared with the reference support obtained from only TEOS and APTES (sample 3).

A very important observation is that all the samples which showed a negative value of zeta potential exhibit a lower capacity of immobilizing HA by ionic bonds [5,26]. This is mainly because the HA molecules are electrostatically bounded on the aminosilica supports [9].

## 3. Experimental Section

### 3.1. Materials

Tetraethyl orthosilicate (TEOS), 3-aminopropyltriethoxysilane (APTES), methyltriethoxy silane (MeTES), trimethylethoxysilane (Me_3_ES) and bis(3-trimethoxisilylpropyl)amine (BAPTMS), diethoxydimethylsilane (Me_2_DES), 1,2-bis(triethoxysilyl)ethane (BETES) from Sigma-Aldrich, St. Louis, MO, USA were used as received. Ammonium hydroxide (NH_4_OH 25% solution) (Chimreactiv, Bucharest, Romania) and ethanol (Chimopar, Bucharest, Romania) were used without further purification. The commercial sort of humic acid (AHA), used for testing the coupling efficiency of the amino-silica supports, was purchased from Aldrich. The other sort of humic acid (PHA) was extracted in the laboratory from a local peat deposit as in reference [27].

### 3.2. Characterization Techniques

The structure and morphology of the synthesized amino-silica particles were characterized by CP/MAS, NMR, FTIR, elemental analysis (CHN), DLS, LDV, TGA, TG coupled with MS, ESEM and N_2_ adsorption-desorption technique. The coupling efficiency of humic acids on the aminosilica supports was evaluated by UV-VIS technique.

Elemental analysis (CHN)—Elemental composition was determined using a Thermo FlashEA^®^ 1112 analyzer (PerkinElmer Instruments LLC, Shelton, CT, USA).

Fourier transformed infrared spectroscopy (FTIR): Samples were analyzed as pellets (0.001 g of silica sample diluted in 1 g KBr), by transmission FTIR, using a Tensor 37 (Bruker, Munich, Germany). The measurements were recorded in the 4000–400 cm^−1^ range.

Solid State Nuclear Magnetic Resonance (ssNMR): All the NMR spectra were recorded at room temperatures (~303 K) on a Bruker Avance II 400 spectrometer (Bruker, Munich, Germany), with proton frequency of 400.13 MHz (9.38 T) equipped with a 4 mm CP/MAS probes. All solid-state experiments included ramped-amplitude cross polarization and magic-angle spinning. The contact time varied between 2 and 3 ms. The relaxation delays were 2–4 s, and the number of scans varied between 2 and 4 k. Between 100 and 120 mg of sample was used to fill up the zirconia rotors. The ^13^C spectra were recorded under high-power proton decoupling (SPINAL-64 spin decoupling scheme). The rotation speed varied between 3000 and 6000 Hz for the ^29^Si and ^13^C nuclides, respectively. For the measurements we have used standard NMR methods (Software: Bruker Biospin Topspin 2.1.3., Bruker, Munich, Germany): C-13 and Si-29 variable amplitude CP/MAS. The chemical shifts were externally referenced to glycine and tris(trimethylsilyl)silane (TTMSS).

The particle hydrodynamic medium diameter and size distribution were measured by DLS (Dynamic Light Scattering) technique in aqueous dispersion (0.02 g of dry sample was re-dispersed in 25 mL distilled water, using a ultrasonic bath for 10 min.), employing Zetasizer Nano ZS ZEN 3600 instrument, manufactured by Malvern Instruments Ltd. (Malvern, UK). The device has a “red” He-Ne laser (633 nm) and can perform measurements of particle size in the range 0.6 to 6000 nm. Disposable polystyrene cells DTS0012 (Malvern Instruments Ltd., Malvern, UK) were used.

The same instrument, Zetasizer Nano ZS ZEN 3600, was used to perform zeta potential measurements, based on electrophoretic mobility measurement technique, LDV (Laser Doppler Velocimetry). Disposable cell DTS 1060 (Malvern Instruments, Malvern, UK) were used. The same dispersions prepared for DLS measurements were also used for zeta potential analysis.

ESEM images of silica supports were obtained with a scanning electron microscope FEI Quanta 200 (Philips, Eindhoven, The Netherlands). The samples were dispersed in distilled water (0.02 g of milled sample in 25 mL of distilled water), deposited on the corresponding sample carrier by dropping with a pipette and let them dry in air.

The nitrogen adsorption-desorption isotherms were recorded at −196 °C, using a NOVA 2200e Automated Gas Sorption instrument (Quantachrome Instruments, Hartley Wintney, UK). Prior to measurements, samples were degassed under vacuum at 100 °C for 5 h. The Brunauer–Emmett–Teller specific surface areas of samples were calculated from adsorption data at a relative pressure range of 0.08–0.3. The total pore volumes were evaluated from the adsorbed amount of nitrogen at a relative pressure of 0.986.

Thermogravimetric Analyzes were performed in air (60 mL/min), using a SDTA 851^e^Mettler Toledo instrument (Mettler-Toledo GmbH, Schwerzenbach, Switzerland). The TG diagrams were measured in the 25–700 °C range with an increment of 10 °C/min.

In order to check contamination of the resulted aminosilica supports with atmospheric carbon dioxide, TG-MS measurements were also performed. A thermoanalyzer (TA instruments, New Castle, DE, USA) combined with a quadrupole mass spectrometer (Pfeiffer Vacuum, Aßlar, Germany) by a capillary inlet system allowed simultaneous DTA, TG and evolved gas analysis of samples. Thus, thermogravimetric analyses were performed in helium with a SDT Q600 instrument (TA Instruments, New Castle, DE, USA), module DSC-TGA (standard sample size: 9.5–10.5 mg; pan: alumina; purge gas: helium 5.0, 100 mL/min; method: ramp 10 °C/min to 900 °C), while for the evolved gas analysis (EGA) a Quadrupole Mass Spectrometer ThermoStar TM GSD 301 T instrument (Pfeiffer Vacuum, Asslar, Deutschland) was used (quartz glass capillary, heated up to 200 °C).

The capacity of aminosilica supports to capture the HA molecules was evaluated by UV-VIS spectroscopy. The absorbance measurements for the filtered dispersions were recorded with a Nicolet Evolution 500 instrument (Thermo Electron Corp., Waltham, MA, USA). A buffer solution having pH = 7.5 was prepared from 700 mL KH_2_PO_4_ 0.1M and 575 mL of NaOH 0.1M. Thereafter, 100 mL of stock solutions of humic acids in buffer were prepared at a concentration of 1 g/L. The resulted stock solutions were further used for the acid immobilizing tests. The results shown in Figure 9 are the average of 3 measurements for each sample. All the materials subjected to the humic acid (HA) coupling tests were previously milled. Accurately weighed quantities (0.5 g) of the milled silicas were added to 5 mL of HA stock solution and were left in contact for 20 h. Then, by means of syringes equipped with 0.2 μm filters, 1 mL of solution was collected from the supernatant of each sedimented sample and the absorbency was recorded by UV-VIS technique at a fixed wavelength (254 nm) [9]. Concentrations of the HA remained in the filtered solution were calculated from the measured absorbencies and by means of the equations resulted from the corresponding calibration curves. Subtracting the concentration of the free HA from the initial concentration of HA present in the stock solution resulted in the concentration of HA chemically attached onto the silica supports.

### 3.3. Synthesis of the Silica Hybrid Supports

A method adapted from reference [1] was used for the synthesis of the amino-functionalized silica particles, through a two-step sol-gel method.

Step 1.  Preparation of Pristine Silica Particles (Preformed SiO_2_ Particles)

Forty milliliters of ammonia and 200 mL of ethanol were introduced in a flask equipped with a mechanical stirrer, condenser and addition funnel. 10 g (0.048 moles) of TEOS diluted in 40 mL of ethanol were rapidly added to this mixture and kept under stirring (~200 rot/min) for another two hours at room temperature.

Step 2.  Preparation of Amino-Functionalized Silica Particles (NH_2_-SiO_2_ Particles)

Over the dispersion prepared in Step 1, 0.532 g of APTES (0.0024 moles) diluted in 4 mL ethanol were continuously added for two hours. This amount represents an APTES/TEOS molar ratio of 1/20. The co-precursors/TEOS molar ratio was chosen according to the method described by Brambilla *et al.* [28]. The surface modification of the preformed SiO_2_ particles was previously performed by other groups [29,30], but our preparation procedure is different from what was previously reported. Thus, Jung *et al.* [29] obtained amino-functionalized silica particles only in ethanol medium and after the re-dispersion of the pristine SiO_2_ particles. Also, Meera *et al.* [30] prepared castor oil based polyurethane (PU)–silica nanocomposite films by adding amino functionalized silica particles to the polyurethane mixture. Their amino functionalized silica particles were prepared by a method similar to that used by us, but they did not study the effect of the alkyl spacers over the particles ability to immobilize humic acids.

In some cases, the alkyl derivatives (spacers for the amino functions)—methyltriethoxy silane (MeTES), trimethylethoxysilane (Me_3_ES), diethoxydimethylsilane (Me_2_DES) and 1,2-bis(triethoxysilyl) ethane (BETES)—were added together with APTES to the reaction system. The final mixture was further stirred for two more hours and then allowed to rest overnight. Subsequently, one part from the resulted dispersion was kept in a closed bottle and another part has been transferred to a Petri dish and allowed to dry at room temperature for 3–4 d. Finally it was dried under vacuum at 40 °C.

For preparation of sample 1 (only APTES), 10.6 g APTES diluted in 40 mL EtOH were introduced over a mixture of 40 mL NH_4_OH 25% and 200 mL EtOH. The reaction was kept under stirring for 4 h and left over night. The next day the whole mixture was a homogenous and transparent dispersion. After the removal of the volatile compounds, the resulted solid was transparent and difficult to be milled. This reference sample was prepared in order to demonstrate the effect of sterical hindrance over the ability to immobilize the HA, as a result of the high density of the amino groups.

## 4. Conclusions

Amino-silica supports for humic acid immobilization were developed by grafting of the amino functions (alone or spaced by alkyl functions) onto preformed mono-dispersed SiO_2_ particles (549 nm). Zeta potential measurements showed that, by increasing the APTES weight share in the reaction system, negative values of zeta potential were obtained. Due to their hydrophobic character, the aminopropyl chains tend to self-associate and to orientate towards the interior of the silica particle (a water repellent behavior). By this phase-inversion mechanism, the -NH_2_ functions are partially hidden inside the silica frame, while towards the water are predominantly exposed Si-O^−^ charges, lowering the ability of the resulted material to interact with HA molecules. Meanwhile, all the APTES/TEOS = 1/20 hybrid materials, having a low density of the free aminopropyl functions on the particles surfaces, showed positive values of zeta potential and were able to efficiently interact with external large HA molecules, as confirmed by the immobilization tests.

The presence of the aminopropyl chains and methyl, dimethyl and ethylene moieties on the silica surface was evidenced using ^29^Si and ^13^C ssNMR spectroscopic methods. The type of bonded silyl species (-O_4_Si, -O_3_SiR, -O_2_SiR_2_), resulted by the organosilanization reaction, was confirmed by ^29^Si CP/MAS technique. The ^13^C CP/MAS spectra proved that the organic groups are indeed present in the relevant samples. Integration of the bi- and tri-functional alkyl silanes (Me_2_DES and MeTES) in the silica frame was also confirmed by the properties change of the final particles (both sample exhibited a strong water repelling behavior—the resulted silica powders were floating on the water). It was also evidenced that, perhaps for sterical reasons and for its lower reactivity (only one Si-OEt reactive group), the monofunctional derivative (Me_3_ES) did not connect to the silica network.

The HA immobilization tests revealed that the density of aminopropyl functions is low enough at this low APTES fraction (APTES/TEOS = 1/20) not to satirically hinder the HA complex. In this condition, the spacing of the amino groups by alkyls was successfully achieved, but no significant differences in the HA immobilization capacity were observed.

The structure and properties of synthesized aminosilica materials showed the potential of these novel supports to be successfully applied for the immobilization of different bio-molecules (as was demonstrated here for humic acids).
